# Is Animal Welfare an Internationally Understood Concept in the Zoo World? Thematic Analysis of Two Regional Groups of Zoo Staff

**DOI:** 10.3390/ani11072059

**Published:** 2021-07-10

**Authors:** Heather Bacon, Belinda Vigors, Darren J. Shaw, Natalie Waran, Cathy M. Dwyer, Catriona Bell

**Affiliations:** 1 Royal (Dick) School of Veterinary Studies, The Roslin Institute, Easter Bush Campus, University of Edinburgh, Roslin EH25 9RG, UK; Darren.Shaw@ed.ac.uk (D.J.S.); cathy.dwyer@ed.ac.uk (C.M.D.); 2 School of Veterinary Medicine, University of Central Lancashire, Preston PR1 2HR, UK; 3 Department of Animal Behaviour and Welfare, Scotland’s Rural College (SRUC), West Mains Road, Edinburgh EH9 3RG, UK; Belinda.Vigors@sruc.ac.uk; 4 Eastern Institute for Technology, 501 Gloucester Street, Taradale, Napier 4142, New Zealand; nwaran@eit.ac.nz; 5 Advance HE, Holyrood Park House, 106 Holyrood Road, Edinburgh EH8 8AS, UK; catriona.bell@advance-he.ac.uk

**Keywords:** zoo, welfare, education, China, Europe

## Abstract

**Simple Summary:**

In order to ensure we do not speak at cross-purposes, common understandings and definitions are useful. However, there is no universal definition of animal welfare. Structured interviews with a sample of European and Chinese zoo staff aimed to explore their perceptions and understanding of the term ‘animal welfare’, and the use of animal welfare frameworks in a zoological context. Thematic analysis demonstrated that all interviewees used similar descriptors when discussing animal welfare including describing animal feelings and emotions. Animal welfare frameworks were considered useful across both regions. However, different frameworks were suggested by interviewees in Europe to those in China. Chinese zoo staff expressed the importance of leadership in animal welfare in Chinese zoos. These findings suggest a common understanding of the concept of animal welfare, and the usefulness of animal welfare frameworks across geographically and culturally diverse regions.

**Abstract:**

Universal frameworks for zoo animal welfare have been suggested. However, there is little evidence of a cross-cultural understanding of zoo animal welfare. This paper reports themes emerging from a qualitative study of international (European and Chinese) zoo professionals on zoo animal welfare issues. Structured telephone interviews were conducted with eight Chinese and eight European zoo staff, covering aspects of zoological animal welfare, conservation and zoological husbandry practices. These qualitative data were thematically analysed, and key themes generated. This paper describes three themes relating to ‘What is animal welfare’ ‘Animal welfare frameworks’ and ‘The human element in animal welfare’. This analysis indicates that the concept of animal welfare has cultural equivalence across Europe and between Europe and China, and that zoo staff are familiar with welfare frameworks. In China, a need for senior leadership and motivating staff to improve animal welfare emerged.

## 1. Introduction

Animal welfare is an important area of research to the global zoo community [1,2]. However, a number of gaps still exist both in terms of understanding the welfare needs of zoo animals [1] and also in terms of addressing those gaps effectively through collaboration, education and mentoring [3]. An extra challenge is added when we consider the international cross-cultural nature of the global zoo community and the different ways in which different people perceive and value animal welfare in different contexts [4,5]. Guidance and educational resources in animal welfare for zoo professionals are increasing, e.g., Wild about Welfare interactive resource https://wildwelfare.org/resources-elearn-programme/ (accessed on 19 April 2021), webinars and welfare assessment models https://www.eaza.net/about-us/areas-of-activity/animal-welfare/ (accessed on 24 February 2021) but these resources are developed primarily by Western organisations on the assumption that there is an international understanding of zoo animal welfare.

Several different animal welfare frameworks have been developed, starting with the Five Freedoms framework developed by John Webster in in 1993/1994 [6,7] and based on the findings of the Brambell committee, a UK government report on the welfare of intensively farmed livestock [8]. Since then, a number of other welfare frameworks have been developed in other industries and applied to zoo animals; these include the Welfare Quality © [9,10], Five Needs/Provisions [11] and Five Domains [12,13] models. Universal approaches to education may not be effective, and an understanding of the target audience has been suggested to be important [14,15]. However, the understanding and acceptance of different welfare frameworks outside of the Western world have not been well explored and there is a lack of literature as to what the international zoo community understands by animal welfare, and what they feel is important in terms of providing good zoo animal welfare. Without a better understanding of the baseline perceptions of the zoo community towards animal welfare, we cannot be assured that universal guidance on animal welfare, or educational interventions targeted at zoo staff from different cultural and geographic backgrounds are suitable.

Political and cultural barriers to accessing zoo animal welfare resources also exist [16]. For example, whilst the Five Domains framework has been adopted by the World Association of Zoos and Aquaria (WAZA) [12], because of political challenges, and the published literature language bias, many zoos in China remain outside of WAZA membership (https://www.waza.org/members/waza-members/, accessed on 19 April 2021) and thus potentially isolated from international guidance and research [17]. Additionally, it is unclear whether the Five Domains framework is well understood internationally, as it may be misapplied even in English-speaking contexts where access to the research is easier, e.g., Section 5 of the Irish standards of modern zoo practice [18]. Limited work performed to date across international zoo communities indicates that specific issues relating to animal welfare (animal behaviour, positive animal mental states and human health and safety) may be of common concern to zoo staff, but the inclusion of health and safety raises the question of whether the concept of animal welfare is universally understood across different cultures [16]. With these potential challenges in mind, it is important to characterise the ‘status quo’ of the understanding of zoo staff towards animal welfare before we can develop effective education or encourage the universal application of animal welfare assessment frameworks.

Two study areas were selected to explore the similarities and differences in the perceptions of zoo staff to zoo animal welfare: (1) Europe—a culturally and linguistically diverse region where a significant volume of animal welfare literature and legislation has been generated [19] and where the regional zoo association provides clear leadership, research and education on zoo animal welfare [1,20,21],; (2) China—a more culturally and linguistically homogeneous region with linguistic, cultural and political separation from international zoo associations, animal welfare research and education. The significant variation between and (in the case of Europe) within these two regions provides an opportunity to elucidate and characterise any differences in perceptions of what animal welfare is.

The aim of this study was to investigate whether the term ‘animal welfare’ has equivalence in culturally diverse zoo communities with differing access to zoo animal welfare education and resources. Study regions were set as Europe and China, and the objective was to explore perceptions of European and Chinese zoo staff to concepts of animal welfare using structured interviews, and to identify key emergent themes using a thematic analysis approach.

## 2. Materials and Methods

Ethical approval for this project was obtained from the Royal (Dick) School of Veterinary Studies Student survey group, at the University of Edinburgh. This paper reports results from a larger research project aiming to characterise the training needs of zoo staff in Europe and China on the topics of animal behaviour and welfare and to identify potential barriers to improving welfare in zoos.

Structured interviews were selected to provide clarity of terminology when translated and to minimise the potential for confusion of terminology or context of questions in a diverse international sample. However, open and discursive responses were encouraged from interviewees. The interview script was informed by a review of the literature, and the outcomes of surveys of an international sample of zoo staff attending either an educational workshops (n = 73, face-to face survey) or completing a massive open-access online course (MOOC) (n = 30, online survey). These data were triangulated and informed the content of the interview script. The interview script was structured in three sections: 1. demographics and zoo perceptions, 2. animal welfare knowledge and education, and 3. controversial zoo practices (Supplementary S1).

A sampling matrix (described in [22]) was used to ensure a maximum purposive sample across a range of job roles in the zoo. Purposive sampling is used to ensure that the interviewees selected have knowledge of the interview subject and reflect a broad range of experiences [23]. Interviewee inclusion criteria included that interviewees must be working in a zoo in Europe (EU1–EU8) or a zoo in the People’s Republic of China (CN1-CN8) and employed in one of six job roles influencing animal care (keepers, team leaders/senior keepers, technical (e.g., biologist, training coordinator) veterinarian, management/curator, director/senior management). Interviewees were recruited from professional networks by request. All interviewees gave informed consent to participate in the project. The Chinese interview script was back-translated and the script was piloted with a Chinese and a European zoo keeper. Piloting ensured the script was clear and covered the topics of interest. Based on piloting, the interview scripts were refined to reduce question numbers as the interview duration was over 40 min, and minor edits were made to the script to reduce the need for any verbal clarification of questions, but the content of the interview script was not substantively changed after piloting. The interviews with European participants were delivered by telephone in English and the data transcribed directly in English. Chinese interviews were conducted via a translator who read from a Chinese script directly to the Chinese interviewee and verbally back-translated their responses into English.

Interviews were conducted as described above and whilst the script was followed, interviewees were encouraged to expand on points of interest in line with a structured ethos. Recorded interview responses were transcribed professionally (University Transcriptions, TP Transcription Limited, UK) and responses were cleaned, with contextual information added in square brackets to ensure clarity of meaning. At no time was the meaning of the text changed, nor were any errors in grammar or syntax corrected. Transcribed interview data were cross-checked against the original audio recordings for accuracy.

European and Chinese datasets were analysed separately. Each interview script was coded using NVIVO 11 (QRS) with both a priori codes derived from research questions, and coding of emergent themes arising from the decontextualised interview data. Each dataset was then coded by interview question (across case) to compare responses between interviewees. Interviews continued until saturation occurred (no further themes emerged). The pilot interview data were analysed last, and the responses found to be consistent with other responses within their datasets and so were included within the sample.

## 3. Results

Eight interviewees were interviewed from each of the two regions (China and Europe) for a period of 25–45 min. Demographic information has been reported separately [22] but interviewees were spread across six cities in China and seven cities (six countries) across Europe. All questions were answered by all interviewees. Thematic analysis inductively identified twelve overarching themes. The themes relating to interviewees understanding and interpretation of animal welfare in zoos are reported below with illustrative quotes.

### 3.1. Theme 1: A Universal Understanding of Zoo Animal Welfare (EU and CN)

Results from this study indicate that there is cultural equivalence in the understanding of zoo animal welfare between zoo staff in Europe and in China. A common understanding of a concept is important in ensuring that staff are working together to achieve progress in that area, and interviewees from both regions using similar descriptors and definitions of animal welfare with most touching upon elements of physical, behavioural, emotional and psychological well-being.

*“It’s meeting their needs on a multitude of levels, behavioural, health, psychologically, emotionally.”* (EU4)

*“We need to provide enough food and water for them to live happily in this environment and create an environment that is similar to the animal’s natural habitat, and no disease”* (CN6)

The emotional aspect of welfare including words such as ‘happy’ was mentioned by interviewees in both regions, which is interesting as emotions or ‘feelings’ as a component of animal welfare can still be controversial in some industries.

Increasingly, the importance of choice and control is recognised in supporting good animal welfare [24,25,26,27], and this was also a key element of interviewee’s descriptions of animal welfare, and suggested to be important by interviewees from both regions.

*“…they should have the ability to choose, to have a free choice as much as possible”* (EU2)

*“to let the animals live wherever they like and ensure enough food and water, improve their mental health by providing the animals as much as possible choices to let them have different experiences”* (CN1)

Interviewees from both regions also mentioned both positively and negatively valanced aspects of an animal’s welfare experience, including mental and physical aspects, but also touching upon some welfare indicators such as abnormal repetitive behaviours, and behavioural management strategies that may be employed to mitigate or prevent welfare problems:

*“the most important thing is to provide a natural environment for the animal if the zoo cannot do this they should try other ways reduce the abnormal behaviours of the animals”* (CN2)

*“the individual state of an animal and its ability to experience positive and negative state, as well as sort of, I suppose, a capacity for suffering experiencing pleasure”* (EU6)

In short, interviewees from both regions described animal welfare in terms of physical, mental, emotional and behavioural well-being, acknowledged that welfare could be both positively and negatively valanced, and discussed the importance of choice for zoo animal welfare. This common understanding aligns with the published literature in animal welfare [27,28,29,30] and whilst no universal definition of animal welfare exists [31], these elements are well established within the various published animal welfare definitions and frameworks.

### 3.2. Theme 2: Frameworks (EU and CN)

The ‘Five Freedoms’ framework was freely elicited by interviewees from both regions as being useful to evaluating animal welfare, highlighting its international applicability to the concept of animal welfare.

*“I think it’s providing animals with physical, social, psychological environment that is consistent with their natural needs and avoids, it’s the five freedom thing. It’s freedom from fear, and pain and distress”* (EU7)

*“Thinking of 5 freedoms but can’t think of all of them, most important thing is to make animals happy and enable them to have enough choices”* (CN4)

Whilst no interviewee volunteered to recite all of the Five Freedoms, all interviewees except one (EU8) were familiar with the Five Freedoms as a framework for animal welfare. In China, the Five Freedoms were considered to be an aspirational standard by one interviewee.

*“…in China it is only a wish to accomplish the 5 freedoms.”* (CN5)

Conversely, the Five freedoms were considered not to go far enough by half of the European respondents; whilst all interviewees thought that animal welfare frameworks are useful in the management of zoo animals, four of the European interviewees felt that the Five Freedoms were too limited as a framework for zoo animal welfare.

*“I think it’s wider. The welfare is wider than five freedoms.”* (EU5)

*“I don’t think they go far enough but it’s good to at least have a very basic starting point and then you can build from there, other more advanced framework now, like the 12 animal welfare assessment criteria”* (EU6)

*“I think it can have its limitations, but I think it’s a really good back bone to something actually black and white that can be followed”* (EU4)

More recent literature in zoo animal welfare has often promoted alternative frameworks such as the ‘Welfare Quality’ approach (12 welfare assessment criteria) [9,10] or the ‘Five Domains’ model [12,32]. It appears that these more recent frameworks may be less familiar to zoo staff, as they were not spontaneously suggested as frequently as the Five Freedoms were.

There are differing perspectives on the value and use of animal welfare frameworks both within and between the study regions but all interviewees felt that frameworks were helpful in providing a structure to assessing zoo animal welfare.

### 3.3. Theme 3: The Human Element (CN)

All interviewees agreed that consideration of animal welfare was relevant to successful zoo animal husbandry, but the impacts of animal welfare on the human community was raised by Chinese interviewees only. The interviewees discussed the importance of encouraging and motivating staff, and leadership support for animal welfare, a consideration that did not arise from the European discussions on animal welfare.

*“If the management do not recognize animal welfare, then the animal welfare can not be improved, and only if everyone reaches agreement, can we do things well.”* (CN8)

*“the leaders including the forestry bureau have no idea about animal welfare, while they do not consider these when evaluating work. This work can only be carried out based on zoo’s self-consciousness.”* (CN7)

The lack of this theme from European interviewees could suggest that they feel that leadership encouragement is not relevant to providing good animal welfare, or that delivering good animal welfare is more dependent upon personal responsibility than management support or it may be that support for animal welfare is already an inherent element of European zoo animal management. This suggestion is supported by the EAZA who state “*EAZA is committed to promoting the positive welfare of animals, not only in our member institutions but also through supporting zoos and aquaria which are currently working towards reaching EAZA’s accreditation standards*” (https://www.eaza.net/about-us/areas-of-activity/animal-welfare/, accessed on 24 February 2021). It may be that animal welfare in Chinese zoos does not yet have such leadership support. Whilst the Chinese Association of Zoological Gardens (CAZG) published an Ethics and welfare statement in 2012, it focuses on quite basic practices and safeguards. These include participating in wildlife rescue, forbidding animal abuse and providing ‘good living conditions’ (including space, environmental enrichment, safety facilities and medical care), rather than an aspirational approach to championing good zoo animal welfare [33]. The importance of staff engagement with animal welfare was emphasised by one Chinese interviewee, indicating that animal care staff may feel a burden of responsibility to animal welfare that is not supported by more senior roles.

*“Welfare is mostly created by animal management staff”* (CN2)

However, it may also be that animal care staff sometimes also do not really understand the importance of good animal welfare and feel ‘forced’ into engaging in animal welfare activities. One Chinese interviewee suggested that more emphasis on the connection between animal and human well-being may be important in motivating improvements in animal welfare in China.

*“the improving animal welfare also have positive influence on zoos and human beings, so we should actively improve animal welfare rather than being forced to do it.”* (CN7)

These quotes emphasise the importance of staff engagement with animal welfare and their understanding of the human dimension of animal welfare. It appears that there is possibly conflict around levels of knowledge of animal welfare and its importance across zoos in China.

## 4. Discussion

This paper reports the three themes relating to zoo animal welfare that emerged from interviews with a sample zoo staff from geographically and culturally diverse regions of the world. Whilst the literature on zoo animal welfare has increased over recent years [1], to date there is a gap in the literature regarding how animal welfare is defined and perceived by the international zoo community. This paper begins to address that gap by analysing the perceptions of zoo staff in Europe and China on zoo animal welfare. A limitation of this study is its reliance on English-speaking European interviewees, so whilst a purposive maximum variation geographic sample was used, interviewees were all educated to university level, which may limit generalisability of findings. Additionally, further work is needed to explore other international samples.

There are many definitions and theoretical models of animal welfare, e.g., [28,34,35], and because animal welfare is a multidisciplinary field comprising both social and natural sciences, understandings of what animal welfare means may vary depending on culture and context [36]. A universal framework for zoo animal welfare has been suggested [37] and the World Association for Zoos and Aquaria has produced a global strategy for zoo animal welfare [12], but it is not yet established as to whether there is a global understanding of animal welfare as a concept. However, the findings in this paper suggest that at least for Europe and China, there is a cultural equivalence as to the meaning of the term ‘Animal Welfare’. Cultural equivalence is when respondents assign the same meaning to a concept regardless of their cultural linguistic group [38,39]. It is important to ascertain whether cultural equivalence exists prior to engaging in educational activities as without it, it would be easy for participants to discuss the same topic at entirely cross-purposes. Interviewees from both regions recognised the emotional/affective (animal feelings) dimension of animal welfare, and the importance of providing animals with choice and control [25,27,40].

What did differ between regions was the value of different animal welfare frameworks. All interviewees from both regions except the EU8 were familiar with the Five Freedoms as a framework, and many interviewees from both regions spontaneously volunteered this framework within their description of animal welfare. However, Chinese interviewees did not spontaneously discuss other animal welfare frameworks when specifically asked about frameworks later in the interview, in the way that European interviewees did, possibly indicating that they are less familiar with alternative animal welfare frameworks. This may be because the Five Freedoms are established internationally in a way that newer frameworks have not yet accomplished, and have been used extensively in charitable educational resources internationally, e.g., World Society for the Protection of Animals (now World Animal Protection), ‘Concepts in Animal welfare’ [41]. It was evident that at least half of the European respondents felt that the Five Freedoms were limited as a framework for providing good zoo animal welfare, and that newer concepts were considered more appropriate in promoting ‘positive’ or ‘good’ animal welfare. Positive animal welfare is an increasing focus of zoo animal welfare research [2,27,42], but it may be that frameworks incorporating this concept have yet to filter out globally, although the capacity for positive emotional affect in animals was suggested by interviewees from both regions. It is also interesting that the only interviewee who was not familiar with any animal welfare frameworks was European (Polish but working in Greece), which indicates that there may be variation in familiarity with different animal welfare concepts and frameworks within Europe.

The last theme generated was unique to Chinese interviewees and focussed on the need for institutional and national leadership in the promotion of animal welfare. This theme is interesting as Chinese zoo staff are actually more likely to consider animal care and protection as key parts of zoo conservation activities, whereas European zoo staff focus more on activities that support biodiversity [22], which indicates that whilst animal protection activities are incorporated into the role and core activities of Chinese zoos [33]. Despite this Chinese zoo staff clearly feel that there is still a lack of support for achieving good welfare in Chinese zoos. Good welfare in zoos has been shown to be important to the Chinese general public [43,44]. Although visitors to zoos in China showed a low understanding of the term ‘animal welfare’, almost 90% of zoo visitors were willing to pay for improvements in animal welfare [44]. However, of this surveyed population, those with higher incomes and more executive careers knew less about animal welfare [44]. Zhao and Wu’s research aligns with the results of this study, which indicates dissatisfaction of zoo staff with executive management and government officials’ knowledge and leadership in promoting good animal welfare. Despite this, the concept of animal welfare appeared consistent across all interviewees regardless of job role in the zoo and including senior staff such as zoo directors. It may be that the need for leadership support in animal welfare is required at a higher level than the internal zoo management such as the government ministries that manage Chinese zoos and safari parks (the ministry of urban and rural affairs and the ministry of forestry, respectively) and this is supported by the comments of CN7 who mentioned this. It could also be that whilst senior zoo staff have a theoretical knowledge of animal welfare, they do not support good animal welfare in practice—this has been shown in other industries with pig farmers in Brazil choosing painful management practices whilst recognising the detrimental impacts this would have on animal welfare [45]. This disconnect between knowledge and action is important as it indicates that there may be practical or logistical barriers to achieving good animal welfare, rather than a lack of knowledge of the subject [46]. A limitation of this study was conducting all of the European interviews in English—this limited European interviewees to those with good English language capabilities (and thus good educational attainment) [22]. This may have contributed to the fact that this theme did not emerge from the European interviewees, and we would suggest that more work is needed in this area.

The importance of inspiring and motivating staff to support good animal welfare also emerged, and the relationship between good animal welfare and human well-being was also suggested. This ‘one welfare’ concept is well recognised in other industries [47], but the importance of animal care staff in ensuring good animal welfare has only recently been described in the zoo world [48,49,50]

These findings suggest that whilst these two regions may show variation in leadership and regulation of animal welfare standards in zoos, the actual understanding of what animal welfare is appears common across both regions. This information may be important in guiding future activities to improve zoo animal welfare—it may be appropriate to focus less on knowledge transfer activities relating to educating zoo staff about what animal welfare is, and more on inspiring leadership or regulatory change to support the on-the-ground knowledge of animal welfare that already exists. Where animal welfare frameworks are applied in zoos to help evaluate animal welfare, it may be important to ensure that zoo staff are familiar with the various frameworks and the benefits and limitations of each.

## 5. Conclusions

This study begins to fill a gap in the published evidence on international understandings of animal welfare in the zoo community, and suggests that a common understanding (cultural equivalence) of the term ‘animal welfare’ exists across Europe and between Europe and China. Further work to evaluate cultural equivalence in other parts of the world is needed. The two different geographic populations interviewed showed different levels of familiarity and placed different values on different animal welfare frameworks, suggesting that a universal approach to applying frameworks to zoo animal welfare may not be appropriate. In Chinese interviewees, a need for leadership on improving zoo animal welfare was emphasised, indicating that despite there being a consistent understanding of what animal welfare is within the zoos, other challenges may exist to supporting good zoo animal welfare in practice.

## Data Availability

The data presented in this study may be available on request from the corresponding author. The data are not publicly available due to privacy and consent restrictions.

## Supplementary Materials

The following are available online at https://www.mdpi.com/article/10.3390/ani11072059/s1. Supplementary S1: Zoo staff background info and interview script.
