# Supplementary material for: Is Animal Welfare an Internationally Understood Concept in the Zoo World? Thematic Analysis of Two Regional Groups of Zoo Staff
Source: Animals (Basel). 2021 Jul 10;11(7):2059. doi: 10.3390/ani11072059 (PMC8300246; doi:10.3390/ani11072059)
Supplement: Supplementary file 1 [file animals-11-02059-s001.zip › animals-1234402-supplementary.pdf]

Zoo staff background info and interview script

Date:_______ **Subject ID:_______** **Interviewer:_____________** **Translator:____________**

Male: Female:

Country/City __

“I’d like to start with some background demographic information please”:

- Age:
- What is your current role within this zoo?
- Have you ever owned a pet?
- If yes, could you tell me which species you’ve owned_________________________
- Could you tell me whether you eat meat , or meat and fish or only fish as part of your diet?

  Do not eat meat or fish

  Eat meat and fish

  Eat fish

“Thank you. Now, moving on to more general questions”:

- How long have you worked in zoos?
- Have you worked at any other zoos before this one?
  - If others which and for how long?
- Can you tell me how you came to work in a zoo, in terms of your background and training?
- Could you describe your current job, giving me an outline of your daily activities and responsibilities?
- Did you visit zoos as a child?
  - If yes what influence did this have on you?
- Did you watch wildlife documentaries as a child?
  - What influence did that have on you?
- What do you think is the purpose of the modern zoo?
- What do you understand by the term ’Conservation’?

- What do you understand by the term ‘Animal welfare’
- Have you ever received any formal training for your job in your zoo?
    - If yes, how was this delivered?
- what type of training do you think would have been useful at the start of your job?
- What do you think should be the main priorities for training of animal keeping staff in your zoo to improve animal husbandry?
- Which staff in your zoo should future training programmes be targeted at?
- Who do you think should be responsible for providing staff training in your zoo?
- Have any staff in your zoo ever received any formal training in animal housing and husbandry?
    - If yes, how was this delivered?
- If no, would you like to receive any?
- Are you familiar with concepts of animal welfare?
    - E.g Five Freedoms if prompt needed
- Do you think concepts of animal welfare like these, are relevant to how zoo animals are managed?
    - why/why not?
- Have you ever received any animal welfare training?
    - If yes what did it involve?
    - If no, would you be interested in receiving any training in animal welfare?
- Here are five methods of delivering animal welfare training. Which would be your preferred option and why?
    - Online using an interactive multimedia programme that you can access anytime
    - By DVD using an interactive multimedia programme that you can access anytime
    - By mobile phone using an interactive App
    - By a series of lectures
    - In a workshop/conference at a fixed time and place
- Why do you not prefer the other 4 options?

- Are there any barriers or limitations to providing animal welfare training to staff in your zoo?
    - If so what are these barriers?
- Is there any programme of animal welfare assessment in your zoo?
    - If yes, how is this done and by whom?
    - If no, are there particular reasons why not?
- Who makes decisions relating to changes in animal husbandry or enclosure design in your zoo?
- Who makes decisions regarding collection planning, collection management and animal breeding in your zoo?
- Does the zoo cull surplus animals?
    - Why/why not?
- Does the zoo provide opportunities for the public to have physical contact with animals?
    - Why/why not?
- Do you use surgical or medical contraception to control animal populations in your zoo?
    - Why/why not?
    - Ok a couple of more controversial questions
- Are live vertebrate prey fed to other animals in your zoo?
    - Why/why not?
- Do you support the use of animals in performances in zoos?
    - Why/why not?
- Do you think euthanasia is acceptable for health or welfare reasons?
    - Why/why not?
- Do you think that abnormal behaviour is a welfare problem for animals in zoos?
    - Why/why not?
- Could you give an example of what you consider to be an abnormal behaviour?
- Thank you for your time, I’d be happy to answer any questions that you have
